# Effects of interactions between common genetic variants and alcohol consumption on colorectal cancer risk

**DOI:** 10.18632/oncotarget.23997

**Published:** 2018-01-06

**Authors:** Nan Song, Aesun Shin, Jae Hwan Oh, Jeongseon Kim

**Affiliations:** ^1^ Cancer Research Institute, Seoul National University College of Medicine, Seoul, Korea; ^2^ Department of Preventive Medicine, Seoul National University College of Medicine, Seoul, Korea; ^3^ Molecular Epidemiology Branch, National Cancer Center, Goyang, Korea; ^4^ Center for Colorectal Cancer, National Cancer Center, Goyang, Korea

**Keywords:** colorectal cancer, gene and environment interaction, single-nucleotide polymorphism, alcohol consumption, case-control study

## Abstract

**Background:**

Genome-wide association studies (GWAS) have identified approximately 40 common genetic loci associated with colorectal cancer risk. To investigate possible gene-environment interactions (GEIs) between GWAS-identified single-nucleotide polymorphisms (SNPs) and alcohol consumption with respect to colorectal cancer, a hospital-based case-control study was conducted.

**Results:**

Higher levels of alcohol consumption as calculated based on a standardized definition of a drink (1 drink=12.5g of ethanol) were associated with increased risk of colorectal cancer (OR=2.47, 95% CI=1.62-3.76 for heavy drinkers [>50g/day] compared to never drinkers; *p*_trend_<0.01). SNP rs6687758 near the *DUSP10* gene at 1q41 had a statistically significant interaction with alcohol consumption in analyses of standardized drinks (*p*=4.6×10^-3^), although this did not surpass the corrected threshold for multiple testing. When stratified by alcohol consumption levels, in an additive model the risk of colorectal cancer associated with the G allele of rs6687758 tended to increase among individuals in the heavier alcohol consumption strata. A statistically significant association between rs6687758 and colorectal cancer risk was observed among moderate alcohol drinkers who consumed between >12.5 and ≤50g of alcohol per day (OR=1.46, 95% CI=1.01-2.11).

**Methods:**

A total of 2,109 subjects (703 colorectal cancer patients and 1,406 healthy controls) were recruited from the Korean National Cancer Center. For genotyping, 30 GWAS-identified SNPs were selected. A logistic regression model was used to evaluate associations of SNPs and alcohol consumption with colorectal cancer risk. We also tested GEIs between SNPs and alcohol consumption using a logistic model with multiplicative interaction terms.

**Conclusions:**

Our results suggest that SNP rs6687758 at 1q41 may interact with alcohol consumption in the etiology of colorectal cancer.

## INTRODUCTION

Alcoholic beverages are classified as carcinogenic agents with sufficient evidence for colorectal cancer by the International Agency for Research on Cancer [1]. Absorbed alcohol is distributed to colonocytes through the blood stream and metabolized to acetaldehyde, also a known carcinogen, by intestinal microbes [2]. Following alcohol consumption, acetaldehyde accumulated in colonocytes could induce multiple carcinogenic effects, such as DNA damage, excessive cell proliferation of the colonic mucosa, and inhibition of folate absorption [3].

Alcohol-induced carcinogenesis is affected by alcohol-metabolizing enzymes, including alcohol dehydrogenase (ADH), acetaldehyde dehydrogenase (ALDH), and cytochrome P450 2E1 (CYP2E1), and by the methylenetetrahydrofolate reductase (MTHFR) enzyme, which plays a crucial role in folate metabolism [2]. Accordingly, previous studies have commonly focused on *ADH1B*, *ADH1C*, *ALDH2*, *CYP2E1*, and *MTHFR* genotypes as modifiers of the association between alcohol consumption and risk of alcohol-related cancers, including colorectal cancer [4]. Nevertheless, evidence for gene-environment interaction (GEI) involving alcohol consumption and colorectal carcinogenesis remains inconsistent or inconclusive [4], suggesting the need for more studies on interaction effects between various genetic polymorphisms and alcohol consumption with respect to colorectal cancer risk.

Genome-wide association studies (GWAS) have identified a number of single-nucleotide polymorphisms (SNPs) that may be involved in colorectal cancer susceptibility; however, none are directly involved in the alcohol metabolism pathway. Several studies have investigated possible GEIs between GWAS-identified SNPs and alcohol consumption [5–9]. We hypothesized that colorectal cancer susceptibility SNPs could interact with alcohol consumption to influence the risk of colorectal cancer. To test this hypothesis, we examined associations of alcohol consumption and GWAS-identified colorectal cancer susceptibility SNPs with colorectal cancer risk in a Korean population. Associations were also evaluated after stratification by alcohol consumption levels to understand whether they were modified by alcohol consumption.

## RESULTS

The characteristics of the study participants are shown in Table 1. Among a total of 703 colorectal cancer cases and 1,406 healthy controls, neither average age (56.4 years in cases and 56.0 years in controls) nor the distribution of sex differed, indicating adequate matching. Colorectal cancer cases had higher proportions of participants with a family history of colorectal cancer (*p*=0.01) or lower education level (*p*<0.01) and who were unmarried (*p*<0.01) or did not exercise regularly (*p*<0.01). Body mass index (BMI) was higher among controls (*p*=0.04). Neither alcohol consumption nor smoking status differed between colorectal cases and controls. We investigated associations between alcohol consumption factors and colorectal cancer risk (Table 2). Although general alcohol consumption status (ever vs. never) was not statistically associated with colorectal cancer risk, higher levels of alcohol consumption based on standardized drink amounts were associated with increased risk of colorectal cancer (*p*_trend_<0.01), especially for heavy drinkers compared to never drinkers (odds ratio (OR)=2.47, 95% confidence interval (CI)=1.62-3.76). Similar associations with colorectal cancer risk were also observed for alcohol consumption levels when categorized by the median (*p*_trend_=0.05) and by tertiles of intake (*p*_trend_<0.01); the highest tertiles of alcohol consumption had a statistically significant association with risk of colorectal cancer (OR=1.50, 95% CI=1.08-2.07). When stratified by sex, similar associations were found among men between the higher alcohol consumption categories and increased colorectal cancer risk; these associations were attenuated among women (data not shown).

**Table 1:** Characteristics of colorectal cancer cases and controls, National Cancer Center of Korea, 2010-2013

Characteristics	Cases		Controls		*P*^a^
	(N=703)		(N=1,406)		
	N	(%)	N	(%)	
Age (years), mean (SD)	56.4	(9.6)	56.0	(9.1)	0.31
Sex					>0.99
Men	480	(68.3)	960	(68.3)	
Women	223	(31.7)	446	(31.7)	
Family history of colorectal cancer					0.01
No	638	(90.8)	1,319	(93.8)	
Yes	65	(9.3)	87	(6.2)	
BMI (kg/m^2^), mean (SD)	23.8	(3.4)	24.1	(2.7)	0.04
Education level					<0.01
≤Middle school	255	(36.3)	197	(14.0)	
≤High school	269	(38.3)	457	(32.5)	
≥College or university	179	(25.5)	741	(52.7)	
Marital status					<0.01
Married	595	(84.6)	1,268	(90.2)	
Unmarried^b^	106	(15.1)	134	(9.5)	
Alcohol consumption status					0.50
Never	210	(29.9)	400	(28.5)	
Ever	493	(70.1)	1,006	(71.6)	
Smoking status					0.64
Never	316	(45.0)	617	(45.0)	
Ever	387	(55.1)	789	(55.1)	
Regular exercise participation					<0.01
No	474	(67.4)	566	(40.3)	
Yes	229	(32.6)	833	(59.3)	

Abbreviations: SD (standard deviation) and BMI (body mass index).

^a^T-test for continuous variables and chi-square test for categorical variables.

^b^Unmarried category includes never married, divorced, separated, and widowed individuals.

**Table 2:** Associations between alcohol consumption and colorectal cancer risk

Alcohol consumption	Case		Control		OR^a^	(95% CI)
	(N=703)		(N=1,406)			
	N	(%)	N	(%)		
General alcohol consumption status						
Never	210	(29.9)	400	(28.5)	1.00	(ref.)
Ever	493	(70.1)	1006	(71.6)	1.04	(0.81-1.33)
Alcohol consumption by standardized drink amounts (g/day)						
Never	210	(32.3)	400	(30.2)	1.00	(ref.)
Light (≤12.5)	186	(28.6)	530	(40.0)	0.84	(0.64-1.11)
Moderate (>12.5 and ≤50)	153	(23.5)	324	(24.4)	1.10	(0.80-1.52)
Heavy (>50)	102	(15.7)	72	(5.4)	2.47	(1.62-3.76)
*P* for trend	<0.01					
Alcohol consumption by median intake (g/day)						
Never	210	(32.3)	400	(30.2)	1.00	(ref.)
Equal to or below median (≤11.5)	175	(26.9)	506	(38.2)	0.84	(0.64-1.10)
Above median (>11.5)	266	(40.9)	420	(31.7)	1.33	(0.99-1.80)
*P* for trend	0.05					
Alcohol consumption by tertiles of intake (g/day)						
Never	210	(32.3)	400	(30.2)	1.00	(ref.)
1st tertile (≤5.6)	111	(17.1)	346	(26.1)	0.78	(0.58-1.05)
2nd tertile (>5.6 and ≤21.8)	138	(21.2)	316	(23.8)	1.07	(0.77-1.50)
3rd tertile (>21.8)	192	(29.5)	264	(19.9)	1.50	(1.08-2.07)
*P* for trend	<0.01					

Abbreviations: OR (odds ratio), CI (confidence interval), and BMI (body mass index).

^a^Logistic regression model adjusted for age, sex, family history of colorectal cancer, BMI, education level, marital status, smoking status, and regular exercise.

Among 30 GWAS-identified SNPs, 15 SNPs including rs6687758, rs647161, rs6983267, rs7014346, rs10505477, rs10795668, rs704017, rs11196172, rs174537, rs174550, rs1535, rs4779584, rs10411210, rs961253, and rs2423279 were validated in our study population (*p*<0.05, Supplementary Table 1). Among those SNPs, rs6687758 at 1q41 (intergenic) and rs4813802 at 20p12.3 had statistically significant interactions with alcohol consumption based on standardized drink amounts (*p*_interaction_=4.6×10^-3^ for rs6687758 and *p*_interaction_=0.02 for rs4813802, Table 3). GEIs with colorectal cancer risk for rs6687758 were also observed with general alcohol consumption status (*p*_interaction_=0.02), alcohol consumption categorized by the median (*p*_interaction_ =7.4×10^-3^), and by tertiles of intake (*p*_interaction_=3.9×10^-3^) (Supplementary Table 2). However, observed GEIs did not surpass the corrected threshold for multiple testing (*p*-value for Bonferroni correction<1.6×10^-3^ and false-discovery rate (FDR) adjusted *p*-value<0.05).

**Table 3:** *P*-value for the effect of interaction between GWAS-identified SNPs and alcohol consumption by standardized drink amounts on risk of colorectal cancer

SNP	Cytogenetic region	Mapped gene	Allele^a^		Alcohol consumption by standardized drink amounts (never, light, moderate, heavy)	
			A1	A2	Raw *p*^b^	FDR-adjusted *p*
rs6687758	1q41	*intergenic*	G	A	4.6×10^-3^	0.14
rs10936599	3q26.2	*MYNN*	T	C	0.35	0.80
rs647161	5q31.1	*C5orf66*	A	C	0.79	0.94
rs7758229	6q25.3	*SLC22A3*	T	G	0.90	0.94
rs6983267	8q24.21	*CASC8, CCAT2*	T	G	0.26	0.80
rs7014346	8q24.21	*CASC8*	G	A	0.33	0.80
rs10505477	8q24.21	*CASC8*	G	A	0.34	0.80
rs10795668	10p14	*LOC105376400*	A	G	0.43	0.80
rs704017	10q22.3	*ZMIZ1-AS1*	G	A	0.38	0.80
rs11196172	10q25.2	*TCF7L2*	A	G	0.21	0.80
rs1665650	10q25.3	*HSPA12A*	C	T	0.50	0.84
rs174537	11q12.2	*MYRF*	T	G	0.67	0.84
rs174550	11q12.2	*FADS1*	T	C	0.64	0.84
rs1535	11q12.2	*FADS2*	A	G	0.64	0.84
rs3802842	11q23.1	*COLCA1, COLCA2*	A	C	0.14	0.80
rs10849432	12p13.31	*intergenic*	T	C	0.42	0.80
rs10774214	12p13.32	*CCND2-AS1*	C	T	0.24	0.80
rs11169552	12q13.12	*ATF1, LOC105369765*	T	C	0.85	0.94
rs7136702	12q13.13	*intergenic*	C	T	0.62	0.84
rs4444235	14q22.2	*intergenic*	C	T	0.67	0.84
rs1957636	14q22.3	*LOC105370507*	C	T	0.98	0.98
rs4779584	15q13.3	*intergenic*	C	T	0.24	0.80
rs9929218	16q22.1	*CDH1*	A	G	0.50	0.84
rs12603526	17p13.3	*intergenic*	C	T	0.89	0.94
rs10411210	19q13.11	*RHPN2*	T	C	0.40	0.80
rs1800469	19q13.2	*B9D2, TGFB1*	G	A	0.25	0.80
rs2241714	19q13.2	*B9D2, TMEM91*	C	T	0.40	0.80
rs961253	20p12.3	*intergenic*	A	C	0.56	0.84
rs4813802	20p12.3	*intergenic*	G	T	0.02	0.31
rs2423279	20p12.3	*intergenic*	C	T	0.83	0.94

Abbreviations: GWAS (genome-wide association study), SNP (single-nucleotide polymorphism), FDR (false-discovery rate), SNP (single-nucleotide polymorphism), and BMI (body mass index).

^a^A1 is the risk and A2 is the reference allele according to NCBI dbSNP.

^b^Logistic regression model including interaction term (additive genotypes for each SNP × alcohol consumption) adjusted for age, sex, family history of colorectal cancer, BMI, education level, marital status, smoking status, and regular exercise.

Associations between SNP rs6687758 and risk of colorectal cancer according to general alcohol consumption status and alcohol consumption levels are shown in Table 4. In the additive model, the G allele of rs6687758 was associated with increased risk of colorectal cancer (OR=1.20, 95% CI=1.02-1.42). When stratified by alcohol consumption levels, risk of colorectal cancer associated with the G allele of rs6687758 increased among individuals who were ever or higher-level drinkers. The association between rs6687758 and colorectal cancer risk was statistically significant among ever (OR=1.35, 95% CI=1.11-1.64), moderate (OR=1.46, 95% CI=1.01-2.11), and higher-level drinkers in the above-the-median group (OR=1.52, 95% CI=1.14-2.04) and the highest tertile of alcohol consumption (OR=1.59, 95% CI=1.12-2.25). After stratification by sex, no GEIs were observed between rs6687758 and alcohol consumption, and the statistically significant association between rs6687758 and colorectal cancer risk among ever or higher-level alcohol drinkers remained only among men (Supplementary Table 3). Although a GEI between rs4813802 and alcohol consumption based on standardized drink amounts was observed, the association between rs4813802 and colorectal cancer risk was not statistically significant regardless of alcohol consumption level (data not shown).

**Table 4:** Associations between additive risk allele of rs6687758 and risk of colorectal cancer stratified by alcohol consumption

Alcohol consumption	RAF		OR^a^	(95% CI)	*P*_interaction_^b^
	Case	Control			
rs6687758 (1q41/intergenic, G/A^c^)					
All	0.30	0.27	1.20	(1.02-1.42)	-
General alcohol consumption status					0.02
Never	0.29	0.27	0.90	(0.65-1.24)	
Ever	0.31	0.27	1.35	(1.11-1.64)	
Alcohol consumption by standardized drink amounts (g/day)					4.6×10^-3^
Never	0.29	0.27	0.90	(0.65-1.24)	
Light (≤12.5)	0.29	0.26	1.26	(0.93-1.70)	
Moderate (>12.5 and ≤50)	0.32	0.26	1.46	(1.01-2.11)	
Heavy (>50)	0.31	0.24	1.71	(0.94-3.14)	
Alcohol consumption by median intake (g/day)					7.4×10^-3^
Never	0.29	0.27	0.90	(0.65-1.24)	
Equal to or below median (≤11.5)	0.30	0.26	1.26	(0.92-1.71)	
Above median (>11.5)	0.31	0.26	1.52	(1.14-2.04)	
Alcohol consumption by tertiles of intake (g/day)					3.9×10^-3^
Never	0.29	0.27	0.90	(0.65-1.24)	
1st tertile (≤5.6)	0.29	0.27	1.14	(0.77-1.70)	
2nd tertile (>5.6 and ≤21.8)	0.30	0.26	1.42	(0.98-2.06)	
3rd tertile (>21.8)	0.32	0.25	1.59	(1.12-2.25)	

Abbreviations: SNP (single-nucleotide polymorphism), RAF (risk allele frequency), OR (odds ratio), CI (confidence interval), BMI (body mass index).

^a^Additive effect by logistic regression model adjusted for age, sex, family history of colorectal cancer, BMI, education level, marital status, smoking status, and regular exercise by alcohol consumption.

^b^Logistic regression model including interaction term (additive genotypes for rs6687758 × alcohol consumption).

^c^Risk/reference allele according to NCBI dbSNP.

## DISCUSSION

In this case-control study, we investigated possible interactions between GWAS-identified colorectal cancer susceptibility SNPs and alcohol consumption. We observed that higher levels of alcohol consumption and the risk allele (G) of the SNP rs6687758 had statistically significant associations with increased risk of colorectal cancer. Furthermore, rs6687758 showed a consistent interaction with alcohol consumption and colorectal cancer and was associated with increased risk of colorectal cancer among ever and higher-level alcohol drinkers, suggesting that alcohol consumption could be a possible effect modifier.

Although previous GWAS have discovered common susceptibility SNPs associated with colorectal cancer risk, those SNPs explain only a small proportion (1-4%) of the genetic heritability of colorectal cancer, suggesting that a considerable amount of heritability remains unaccounted for [10]. We analyzed post-GWAS GEIs to investigate the missing heritability [11]. To date, GEI studies have been limited mainly to candidate genes and specific environmental factors. Therefore, we focused on GWAS-identified colorectal cancer-susceptibility SNPs that had been robustly replicated and that were relatively unstudied with respect to GEI in order to discover novel GEI and achieve better insights into the biological mechanisms underlying colorectal cancer.

A number of studies have evaluated whether variants of candidate genes involved in alcohol metabolism interact with alcohol consumption and colorectal cancer; however, the results have been inconsistent. Several studies have found statistically significant interactions of variants in the *ADH1B* [12], *ALDH2* [13], *CYP2E1* [13, 14], and *MTHFR* [15–18] genes with alcohol consumption and colorectal cancer, whereas other studies found no interactions with *ADH1B* [19], *ADH1C* [19, 20], *ADH7* [12], *ALDH2* [12, 19, 21, 22], and *MTHFR* [23–26] variants. Besides alcohol-metabolism related genes, interactions with alcohol consumption have been reported in polymorphisms of the *PTEN* [27], *XPA* [28], *XPC* [28], *XPD* [28], *GPX1* [29], *NQO1* [30, 31], *PPARγ* [32], *MTR* [33], *XRCC1* [34] genes, but no interactions have been reported for polymorphisms in the *OGG1* [29] or *XRCC1* [35] genes or for haplotypes of *ASE-1, RAI*, and *ERCC1* polymorphisms [36].

A few studies have investigated known colorectal cancer-susceptibility SNPs discovered by GWAS or have conducted a gene-environment-wide interaction study (GEWIS) for possible GEIs related to alcohol consumption and colorectal cancer. One study discovered that SNP rs9929218 at 16q22.1 (*CDH1*) showed an interaction with alcohol consumption in the screening phase, but failed to replicate [5]. Another study reported nominally significant GEIs with alcohol consumption for rs16892766 at 8q23.3 (*EIF3H*), rs719725 at 9p24.1 (*UHRF2*), and rs9929218 at 16q22.1 (*CDH1*) in a case-only analysis and in rs3802842 in both case-only and case-control analyses [6]. However, other studies have found no evidence for GEIs for known colorectal cancer-susceptibility SNPs with alcohol consumption and colorectal cancer [7–9]. On the other hand, a previous GEWIS reported that interactions of rs12870649 at 13q14.1 (*CTNNA3*) and rs9409565 at 9q22.32 (*HIATL1*) with alcohol consumption were associated with colorectal cancer risk [6, 37].

In our analyses, the effects of interactions between SNP rs6687758 and alcohol consumption on colorectal cancer risk were consistently statistically significant regardless of general alcohol consumption status or alcohol consumption level. Rs6687758 was associated with colorectal cancer risk in Europeans [38] as well as in East Asians [39]. We were able to replicate the association between rs6687758 and colorectal cancer risk in our East Asian study population with the same/expected direction of association as the prior study. However, another European-population study tested GEIs between rs6687758 and alcohol consumption and they found no statistically significant result [9]. These deviations may be caused by ethnic differences (European vs. Asian populations) in the type of alcoholic beverages consumed, genetic factors involved in alcohol metabolism and sensitivity, or potential interaction with different environmental factors. In addition, in the European-population study the alcohol variable was categorized as <1, 1 to <28, and ≥28g/day due to the multistep harmonization required to combine data across diverse studies, whereas our study categorized it based on standardized drink amounts, as well as by the median and tertiles of intake.

SNP rs6687758 is located in an intergenic region on chromosome 1q41. Rs6687758 lies 125kb upstream of the *DUSP10* gene, which encodes dual-specificity phosphatase 10 (DUSP10, also known as mitogen-activated protein kinase (MAPK) phosphatase 5 (MKP5)). DUSP10 inactivates other MAPKs, such as extracellular signal-regulated kinase 1/2 (ERK1/2), p38, and c-Jun N-terminal protein kinase (JNK), which are involved in cellular proliferation and differentiation, enhancing the progression of colorectal cancer [40]. However, the inhibition of p38 and JNK activation was attenuated by alcohol-induced cellular responses, including cell migration and invasion [41], and p38 MAPK signaling including DUSP10 expression has been reported to be significantly altered in the hippocampus of chronic alcoholics [42]. This finding may imply that DUSP10 is a tumor suppressor and may be modified by alcohol consumption.

In our study, none of the GEI effects of the 30 GWAS-identified colorectal cancer-susceptibility SNPs with alcohol consumption and colorectal cancer risk remained statistically significant, after accounting for multiple comparisons (i.e., *p*-value for Bonferroni correction<1.6×10^-3^ and false-discovery rate (FDR) adjusted *p*-value<0.05). Furthermore, the GEI between rs6687758 and alcohol consumption was not statistically significant in additional case-only analysis and replication study could not be conducted for the finding. This may be because there were no true interactions between the genetic and environmental factors under study. However, when we conducted an internal cross-validation, the estimated area under the receiver operating characteristic (ROC) curve (AUC) values were 0.74 for the original model and 0.73 for the validation model. This indicated a bias in the C statistic of approximately 1% and, thus, an acceptable model performance. Furthermore, SNP rs6687758 showed a consistent interaction with alcohol consumption, irrespective of general alcohol consumption status or alcohol consumption level whether assessed by standardized drink amounts, by the intake median, or by tertiles of intake, indicating that the finding is unlikely to be changed by analyzing alcohol consumption as a continuous variable.

Another reason for the failure to find interactions after multiple testing could be that the study was underpowered to detect potential GEIs due to its relatively small sample size. For a similar reason, the OR for the association between rs6687758 and colorectal cancer risk among heavy drinkers was not statistically significant. In the additive model, when the genetic OR was set to 1.20 for the G allele of SNP rs6687758, the environmental OR was set to 1.04 for ever drinking status, the minor allele frequency (MAF) was set to 0.20, and the prevalence of ever-drinker status was set to 0.71, we had 32-62% power to detect an OR of 1.3-1.5 for GEIs at a 5% significance level. To achieve 80% power, more than 1,069 cases would be required to detect an OR of 1.5 for GEIs under the same settings. In order to alleviate the power problem and identify potential GEIs on colorectal cancer risk, we relaxed the threshold for statistical significance without correction for multiple comparisons.

There are several strengths in this study. All study participants were recruited from the same hospital and treated as uniformly as possible with the same study protocol related to data and blood sample collection in order to minimize systematic errors caused by bias from the selection of the study population or procedures for gathering genetic and environmental data. Moreover, the entire study population was Korean and thus had an ethnically homogenous genetic background, which should reduce bias caused by population stratification. The novel possible GEI of SNP rs6687758 with alcohol consumption in colorectal cancer needs further study with a larger sample size and/or in other ethnicities to validate the findings.

In conclusion, this study observed a novel possible GEI of SNP rs6687758 and alcohol consumption with colorectal cancer risk. The results indicated that the risk allele (G) consistently increased colorectal cancer risk among ever and heavy alcohol drinkers. Our finding suggests that alcohol consumption may strengthen the genetic effects of rs6687758 on risk of colorectal cancer.

## MATERIALS AND METHODS

### Study population

This hospital-based case-control study was conducted by the Korean National Cancer Center (NCC), as previously described in detail [43, 44]. Briefly, among 1,427 eligible colorectal cancer patients who had been histologically confirmed and had given informed consent to participate in the study between 2010 and 2013, 703 patients were included in the study after applying the following exclusion criteria: failure to contact (N=168), patient refusal to participate after the recruitment (N=189), and incomplete questionnaires and/or insufficient blood samples for genotyping (N=367). Controls were recruited from among healthy individuals who visited a cancer-screening center at the same hospital for a health check-up supported by the National Health Insurance Corporation between 2007 and 2014. After applying the same exclusion criteria above, cases and controls were 1:2 frequency-matched for age (5-year intervals) and sex, leaving a total of 703 colorectal cancer cases and 1,406 controls for the analysis. The study was approved by the institutional review board of the NCC (IRB No. NCCNCS-10-350 and NCC 2015-0202).

### Data collection

At enrollment, face-to-face interviews were conducted by trained interviewers with colorectal cancer cases by using a structured written questionnaire. Controls were asked to complete a self-administered questionnaire and were called by interviewers to confirm the responses, as detailed elsewhere [43, 44]. The questionnaire collected general and lifestyle information regarding family history of colorectal cancer, education level, marital status, and alcohol drinking, smoking, and regular exercise habits. BMI (kg/m^2^) was calculated based on weight (kg) and height (m) measured during a physical examination.

### Assessment of alcohol consumption

The collected alcohol consumption information consisted of drinking status (never or ever), usual frequency at which alcohol was consumed (1/month, 2-3/month, 1/week, 2-3/week, 4-6/week, 1/day, and 2/day), the average number of drinks consumed per drinking occasion by type of alcoholic beverage, specifically beer, soju (a Korean distilled spirit), hard liquor, makgeolli (a traditional Korean rice wine), wine, and other fruit wines. The Korean Genome and Epidemiology Study standardized a single drink of alcohol for the the Korean population as follows: 200ml of 4.5% beer, 50ml of 22% soju and other fruit wines, 30ml of 40% hard liquor, 240ml of 6% makgeolli, and 90ml of 13% general wine, which contained 7.90g, 8.78g, 9.58g, 11.50g, and 9.34g of ethanol, respectively, based on the density of ethanol (0.79g/ml) [45]. The total amount of daily alcohol consumption (g/day) was assessed by summing each beverage-specific amount, which was calculated by multiplying the daily frequency, the number of drinks, and the ethanol content. Alcohol consumption was then categorized as light (≤12.5g/day), moderate (>12.5 and ≤50.0g/day), or heavy (>50g/day) based on standardized drink amounts where 1 drink = 12.5g of ethanol [46] or by the median or tertiles of intake.

### SNP selection and genotyping

As described in detail elsewhere [44], we selected 36 colorectal cancer susceptibility SNPs by reviewing previously published GWAS [38, 39, 47–55]. Genomic DNA was extracted by using a MagAttract DNA Blood M48 kit and BioRobot M48 automatic extraction equipment (Qiagen, Hilden, Germany) and genotyping was performed by using an Agenabio MassArray iPLEX® gold assay (Agena Bioscience, Inc., San Diego, California, United States). In brief, from among 36 originally selected SNPs, a total of 30 colorectal cancer-susceptibility SNPs were included the analysis after exclusion of 4 SNPs that failed to genotype and 2 SNPs that were monomorphic.

### Statistical analysis

For each SNP, the Hardy-Weinberg equilibrium (HWE) test was conducted using the chi-square test among controls, as previously reported [44]. We compared 703 colorectal cancer cases and 1,406 healthy controls to test differences in characteristics using a *t*-test for continuous variables and the chi-square test for categorical variables. The association between variants of SNPs or alcohol consumption and colorectal cancer risk was estimated by using a logistic regression model adjusted for potential confounders. We selected age, sex, family history of colorectal cancer, BMI, education level, marital status, smoking status, and regular exercise that were considered to be associated with colorectal cancer risk based on the association test and literature review as the potential confounders. The full model adjusted for all potential confounders provided a better fit than the unadjusted model (*p*<0.01). To investigate the GEIs, we additionally included a multiplicative interaction term composed of the additive genotypes of each SNP and alcohol consumption factors. For susceptibility SNPs that statistically interacted with one or more alcohol consumption factors on colorectal cancer risk, the association between SNPs and colorectal cancer risk was stratified by alcohol consumption factors to evaluate whether it played a role as an effect modifier. Hypothesis testing was performed overall and stratified by sex and estimated by OR, 95% CI, and *p*-values. Statistical significance was considered to be a *p*-value equal to or less than 0.05. For multiple testing, Bonferroni- and FDR-adjusted *p*-values were also calculated, but the corrections were not applied because none of the associations were significant. All statistical analyses were two-sided and performed by using SAS version 9.4 software (SAS Institute, Inc., Cary, North Carolina, United States).

## SUPPLEMENTARY MATERIALS TABLES









## Abbreviations

(ALDH): Acetaldehyde dehydrogenase
(ADH): alcohol dehydrogenase
(BMI): body mass index
(CI): confidence interval
(CYP2E1): cytochrome P450 2E1
(DUSP10): dual-specificity phosphatase 10
(ERK1/2): extracellular signal-regulated kinase 1/2
(FDR): false-discovery rate
(GEI): gene and environment interaction
(GEWIS): gene-environment-wide interaction study
(GWAS): genome-wide association studies
(HWE): Hardy-Weinberg equilibrium
(JNK): c-Jun N-terminal protein kinase
(MAF): minor allele frequency
(MAPK): mitogen-activated protein kinase
(MKP5): mitogen-activated protein kinase phosphatase 5
(MTHFR): methylenetetrahydrofolate reductase
(NCC): National Cancer Center
(OR): odds ratio
(SNP): single-nucleotide polymorphism
