# Supplementary material for: Effects of interactions between common genetic variants and alcohol consumption on colorectal cancer risk
Source: Oncotarget. 2018 Jan 6;9(5):6391–401. doi: 10.18632/oncotarget.23997 (PMC5814220; doi:10.18632/oncotarget.23997)
Supplement: Supplementary file 1 [file oncotarget-09-6391-s001.pdf]

Effects of interactions between common genetic variants and alcohol consumption on colorectal cancer risk

SUPPLEMENTARY MATERIALS

Supplementary Table 1: Association between GWAS-identified SNPs and colorectal cancer risk.

See Supplementary File 1

Supplementary Table 2: *P*-value for effect of interaction between GWAS-identified SNPs and alcohol consumption on risk of colorectal cancer.

See Supplementary File 2

Supplementary Table 3: Association between additive risk allele of rs6687758 and risk of colorectal cancer stratified by alcohol consumption and sex.

See Supplementary File 3
