# Supplementary material for: Effects of interactions between common genetic variants and alcohol consumption on colorectal cancer risk
Source: Oncotarget. 2018 Jan 6;9(5):6391–401. doi: 10.18632/oncotarget.23997 (PMC5814220; doi:10.18632/oncotarget.23997)
Supplement: Supplementary file 2 [file oncotarget-09-6391-s002.doc]

| Supplementary Table 1. Associations between GWAS-identified SNPs and colorectal cancer risk | | | | | | | | | |
| --- | --- | --- | --- | --- | --- | --- | --- | --- | --- |
| SNP | Cytogenetic  region | Mapped gene | Allele^a^ | | *P*_HWE_^b^ | RAF^b^ | OR^c^ | (95% CI) | *P* |
|  |  |  | A1 | A2 |  |  |  |  |  |
| rs6687758 | 1q41 | *intergenic* | G | A | 0.43 | 0.27 | 1.20 | (1.02-1.42) | 0.03 |
| rs10936599 | 3q26.2 | *MYNN* | T | C | 0.22 | 0.61 | 1.04 | (0.90-1.20) | 0.62 |
| rs647161 | 5q31.1 | *C5orf66* | A | C | 0.94 | 0.29 | 1.31 | (1.12-1.53) | 6.0×10^-4^ |
| rs7758229 | 6q25.3 | *SLC22A3* | T | G | 0.14 | 0.21 | 1.11 | (0.94-1.32) | 0.22 |
| rs6983267 | 8q24.21 | *CASC8, CCAT2* | T | G | 0.22 | 0.58 | 0.81 | (0.70-0.93) | 2.8×10^-3^ |
| rs7014346 | 8q24.21 | *CASC8* | G | A | 0.79 | 0.71 | 0.82 | (0.71-0.96) | 0.01 |
| rs10505477 | 8q24.21 | *CASC8* | G | A | 0.16 | 0.58 | 0.80 | (0.69-0.91) | 1.3×10^-3^ |
| rs10795668 | 10p14 | *LOC105376400* | A | G | 0.73 | 0.39 | 0.80 | (0.69-0.93) | 3.0×10^-3^ |
| rs704017 | 10q22.3 | *ZMIZ1-AS1* | G | A | 0.38 | 0.34 | 1.22 | (1.06-1.42) | 7.7×10^-3^ |
| rs11196172 | 10q25.2 | *TCF7L2* | A | G | 0.52 | 0.73 | 1.20 | (1.02-1.41) | 0.03 |
| rs1665650 | 10q25.3 | *HSPA12A* | C | T | 0.13 | 0.67 | 1.07 | (0.92-1.25) | 0.40 |
| rs174537 | 11q12.2 | *MYRF* | T | G | 0.72 | 0.33 | 0.82 | (0.70-0.96) | 0.01 |
| rs174550 | 11q12.2 | *FADS1* | T | C | 0.67 | 0.67 | 1.18 | (1.01-1.38) | 0.03 |
| rs1535 | 11q12.2 | *FADS2* | A | G | 0.86 | 0.68 | 1.18 | (1.01-1.38) | 0.04 |
| rs3802842 | 11q23.1 | *COLCA1, COLCA2* | A | C | 0.13 | 0.59 | 0.99 | (0.86-1.15) | 0.93 |
| rs10849432 | 12p13.31 | *intergenic* | T | C | 0.66 | 0.82 | 1.10 | (0.91-1.32) | 0.34 |
| rs10774214 | 12p13.32 | *CCND2-AS1* | C | T | 0.56 | 0.58 | 0.92 | (0.80-1.06) | 0.26 |
| rs11169552 | 12q13.12 | *ATF1, LOC105369765* | T | C | 0.51 | 0.67 | 0.97 | (0.83-1.12) | 0.67 |
| rs7136702 | 12q13.13 | *intergenic* | C | T | 0.04 | 0.48 | 0.95 | (0.82-1.09) | 0.45 |
| rs4444235 | 14q22.2 | *intergenic* | C | T | 0.52 | 0.52 | 0.99 | (0.86-1.13) | 0.86 |
| rs1957636 | 14q22.3 | *LOC105370507* | C | T | 0.74 | 0.40 | 1.14 | (0.99-1.32) | 0.07 |
| rs4779584 | 15q13.3 | *intergenic* | C | T | 0.91 | 0.16 | 0.80 | (0.66-0.98) | 0.03 |
| rs9929218 | 16q22.1 | *CDH1* | A | G | 0.47 | 0.15 | 1.15 | (0.95-1.40) | 0.15 |
| rs12603526 | 17p13.3 | *intergenic* | C | T | 0.93 | 0.36 | 0.97 | (0.84-1.13) | 0.72 |
| rs10411210 | 19q13.11 | *RHPN2* | T | C | 0.03 | 0.21 | 0.76 | (0.64-0.92) | 4.1×10^-3^ |
| rs1800469 | 19q13.2 | *B9D2, TGFB1* | G | A | 0.39 | 0.51 | 1.05 | (0.91-1.21) | 0.52 |
| rs2241714 | 19q13.2 | *B9D2, TMEM91* | C | T | 0.38 | 0.51 | 1.02 | (0.89-1.17) | 0.76 |
| rs961253 | 20p12.3 | *intergenic* | A | C | 0.97 | 0.09 | 1.34 | (1.06-1.70) | 0.01 |
| rs4813802 | 20p12.3 | *intergenic* | G | T | 0.81 | 0.21 | 1.02 | (0.86-1.22) | 0.81 |
| rs2423279 | 20p12.3 | *intergenic* | C | T | 0.90 | 0.27 | 1.18 | (1.01-1.38) | 0.04 |
| Abbreviations: GWAS (genome-wide association study), SNP (single-nucleotide polymorphism), HWE (Hardy-Weinberg equilibrium), RAF (risk allele frequency, OR (odds ratio), CI (confidence interval), and BMI (body mass index). | | | | | | | | | |
| ^a^A1 is risk and A2 is reference allele according to NCBI dbSNP. | | | | | | | | | |
| ^b^RAF and *P*_HWE_ were estimated in controls. | | | | | | | | | |
| ^c^Additive effect by logistic regression model adjusted for age, sex, family history of colorectal cancer, BMI, education level, marital status, smoking status, and regular exercise. | | | | | | | | | |
