# Supplementary material for: Effects of interactions between common genetic variants and alcohol consumption on colorectal cancer risk
Source: Oncotarget. 2018 Jan 6;9(5):6391–401. doi: 10.18632/oncotarget.23997 (PMC5814220; doi:10.18632/oncotarget.23997)
Supplement: Supplementary file 3 [file oncotarget-09-6391-s003.doc]

| Supplementary Table 2. *P*-value for the effect of interaction between GWAS-identified SNPs and alcohol consumption on risk of colorectal cancer | | | | | | | | | | |
| --- | --- | --- | --- | --- | --- | --- | --- | --- | --- | --- |
| SNP | Cytogenetic  region | Mapped gene | Allele^a^ | | Alcohol consumption status (never, ever) | | Alcohol consumption  by median intake (never, M1, M2) | | Alcohol consumption  by tertiles of intake (never, T1, T2, T3) | |
|  |  |  |  |  |  |  |  |  |  |  |
|  |  |  | A1 | A2 | Raw *p*^b^ | FDR-adjusted *p* | Raw *p*^b^ | FDR-adjusted *p* | Raw *p*^b^ | FDR-adjusted *p* |
| rs6687758 | 1q41 | *intergenic* | G | A | 0.02 | 0.49 | 7.4×10^-3^ | 0.22 | 3.9×10^-3^ | 0.12 |
| rs10936599 | 3q26.2 | *MYNN* | T | C | 0.45 | 0.92 | 0.30 | 0.81 | 0.23 | 0.70 |
| rs647161 | 5q31.1 | *C5orf66* | A | C | 0.96 | 0.98 | 0.65 | 0.88 | 0.48 | 0.80 |
| rs7758229 | 6q25.3 | *SLC22A3* | T | G | 0.45 | 0.92 | 0.84 | 0.95 | 0.74 | 0.97 |
| rs6983267 | 8q24.21 | *CASC8, CCAT2* | T | G | 0.29 | 0.92 | 0.15 | 0.81 | 0.08 | 0.70 |
| rs7014346 | 8q24.21 | *CASC8* | G | A | 0.31 | 0.92 | 0.21 | 0.81 | 0.30 | 0.70 |
| rs10505477 | 8q24.21 | *CASC8* | G | A | 0.41 | 0.92 | 0.24 | 0.81 | 0.13 | 0.70 |
| rs10795668 | 10p14 | *LOC105376400* | A | G | 0.79 | 0.92 | 0.75 | 0.92 | 0.90 | 1.00 |
| rs704017 | 10q22.3 | *ZMIZ1-AS1* | G | A | 0.69 | 0.92 | 0.56 | 0.84 | 0.57 | 0.86 |
| rs11196172 | 10q25.2 | *TCF7L2* | A | G | 0.20 | 0.92 | 0.15 | 0.81 | 0.16 | 0.70 |
| rs1665650 | 10q25.3 | *HSPA12A* | C | T | 0.21 | 0.92 | 0.39 | 0.84 | 0.30 | 0.70 |
| rs174537 | 11q12.2 | *MYRF* | T | G | 0.74 | 0.92 | 1.00 | 1.00 | 0.97 | 1.00 |
| rs174550 | 11q12.2 | *FADS1* | T | C | 0.72 | 0.92 | 0.98 | 1.00 | 0.90 | 1.00 |
| rs1535 | 11q12.2 | *FADS2* | A | G | 0.62 | 0.92 | 0.18 | 0.81 | 0.93 | 1.00 |
| rs3802842 | 11q23.1 | *COLCA1, COLCA2* | A | C | 0.19 | 0.92 | 0.28 | 0.81 | 0.17 | 0.70 |
| rs10849432 | 12p13.31 | *intergenic* | T | C | 0.30 | 0.92 | 0.41 | 0.84 | 0.33 | 0.71 |
| rs10774214 | 12p13.32 | *CCND2-AS1* | C | T | 0.67 | 0.92 | 0.48 | 0.84 | 0.30 | 0.70 |
| rs11169552 | 12q13.12 | *ATF1, LOC105369765* | T | C | 0.81 | 0.92 | 0.86 | 0.95 | 0.82 | 1.00 |
| rs7136702 | 12q13.13 | *intergenic* | C | T | 0.86 | 0.92 | 0.37 | 0.84 | 0.40 | 0.76 |
| rs4444235 | 14q22.2 | *intergenic* | C | T | 0.79 | 0.92 | 0.62 | 0.88 | 0.53 | 0.84 |
| rs1957636 | 14q22.3 | *LOC105370507* | C | T | 0.50 | 0.92 | 0.56 | 0.84 | 0.63 | 0.89 |
| rs4779584 | 15q13.3 | *intergenic* | C | T | 0.98 | 0.98 | 0.70 | 0.92 | 0.41 | 0.76 |
| rs9929218 | 16q22.1 | *CDH1* | A | G | 0.58 | 0.92 | 0.53 | 0.84 | 0.43 | 0.76 |
| rs12603526 | 17p13.3 | *intergenic* | C | T | 0.77 | 0.92 | 0.95 | 1.00 | 0.68 | 0.92 |
| rs10411210 | 19q13.11 | *RHPN2* | T | C | 0.34 | 0.92 | 0.47 | 0.84 | 0.28 | 0.70 |
| rs1800469 | 19q13.2 | *B9D2, TGFB1* | G | A | 0.51 | 0.92 | 0.17 | 0.81 | 0.13 | 0.70 |
| rs2241714 | 19q13.2 | *B9D2, TMEM91* | C | T | 0.76 | 0.92 | 0.27 | 0.81 | 0.22 | 0.70 |
| rs961253 | 20p12.3 | *intergenic* | A | C | 0.86 | 0.92 | 0.46 | 0.84 | 1.00 | 1.00 |
| rs4813802 | 20p12.3 | *intergenic* | G | T | 0.19 | 0.92 | 0.06 | 0.81 | 0.08 | 0.70 |
| rs2423279 | 20p12.3 | *intergenic* | C | T | 0.64 | 0.92 | 0.77 | 0.92 | 0.84 | 1.00 |
| Abbreviations: GWAS (genome-wide association study), SNP (single-nucleotide polymorphism), FDR (false-discovery rate), SNP (single-nucleotide polymorphism) and BMI (body mass index). | | | | | | | | | | |
| ^a^A1 is risk and A2 is reference allele according to NCBI dbSNP. | | | | | | | | | | |
