# Supplementary material for: Effects of interactions between common genetic variants and alcohol consumption on colorectal cancer risk
Source: Oncotarget. 2018 Jan 6;9(5):6391–401. doi: 10.18632/oncotarget.23997 (PMC5814220; doi:10.18632/oncotarget.23997)
Supplement: Supplementary file 4 [file oncotarget-09-6391-s004.doc]

| Supplementary Table 3. Associations between the additive risk allele of rs6687758 and risk of colorectal cancer stratified by alcohol consumption and sex | | | | | | | |
| --- | --- | --- | --- | --- | --- | --- | --- |
| Alcohol consumption | Men | | |  | Women | | |
|  | OR^a^ | (95% CI) | *P*_interaction_^b^ |  | OR^a^ | (95% CI) | *P*_interaction_^b^ |
| rs6687758 (1q41/intergenic, G/A^c^) | | | | | | | |
| All | 1.44 | (1.17-1.76) | - |  | 0.82 | (0.60-1.11) | - |
| General alcohol consumption status |  |  | 0.42 |  |  |  | 0.42 |
| Never | 1.42 | (0.82-2.48) |  |  | 0.71 | (0.47-1.09) |  |
| Ever | 1.48 | (1.19-1.85) |  |  | 0.91 | (0.56-1.49) |  |
| Alcohol consumption by standardized drink amounts (g/day) |  |  | 0.30 |  |  |  | 0.14 |
| Never | 1.42 | (0.82-2.48) |  |  | 0.71 | (0.47-1.09) |  |
| Light (≤12.5) | 1.45 | (1.01-2.08) |  |  | 0.94 | (0.53-1.67) |  |
| Moderate (>12.5 and ≤50) | 1.49 | (1.02-2.17) |  |  | - |  |  |
| Heavy (>50) | 1.67 | (0.92-3.06) |  |  | - |  |  |
| Alcohol consumption by median intake (g/day) |  |  | 0.26 |  |  |  | 0.16 |
| Never | 1.42 | (0.82-2.48) |  |  | 0.71 | (0.47-1.09) |  |
| Equal to or below median (≤16.2 in men and 2.4 in women) | 1.40 | (0.99-1.98) |  |  | 0.68 | (0.28-1.63) |  |
| Above median (>16.2 in men and 2.4 in women) | 1.60 | (1.16-2.21) |  |  | 1.35 | (0.64-2.85) |  |
| Alcohol consumption by tertiles of inkake (g/day) |  |  | 0.29 |  |  |  | 0.21 |
| Never | 1.42 | (0.82-2.48) |  |  | 0.71 | (0.47-1.09) |  |
| 1st tertile (≤8.2 in men and 1.3 in women) | 1.38 | (0.90-2.12) |  |  | 0.92 | (0.30-2.83) |  |
| 2nd tertile (≤26.3 in men and 4.2 in women) | 1.55 | (1.02-2.36) |  |  | 0.60 | (0.20-1.76) |  |
| 3rd tertile (>26.3 in men and 4.2 women) | 1.62 | (1.09-2.42) |  |  | 1.44 | (0.57-3.68) |  |
| Abbreviations: SNP (single-nucleotide polymorphism), OR (odds ratio), and CI (confidence interval). | | | | | | | |
| ^a^Logistic regression model adjusted for age, sex, family history of colorectal cancer, BMI, education level, marital status, smoking status, and regular exercise by alcohol consumption. | | | | | | | |
| ^b^Logistic regression model including interaction term (additive genotypes for rs6687758 × alcohol consumption). | | | | | | | |
| ^c^Risk/reference allele according to NCBI dbSNP. | | | | | | | |
